# Human laminin-111 and laminin-211 protein therapy prevents muscle disease progression in an immunodeficient mouse model of LAMA2-CMD

**DOI:** 10.1186/s13395-020-00235-4

**Published:** 2020-06-04

**Authors:** Pamela Barraza-Flores, Hailey J. Hermann, Christina R. Bates, Tyler G. Allen, Timothy T. Grunert, Dean J. Burkin

**Affiliations:** Department of Pharmacology, University of Nevada, Reno School of Medicine, Reno, NV 89557 USA

**Keywords:** LAMA2-CMD, MDC1A, Laminin-111, Immune system

## Abstract

**Background:**

Laminin-α2-related congenital muscular dystrophy (LAMA2-CMD) is a devastating genetic disease caused by mutations in the LAMA2 gene. These mutations result in progressive muscle wasting and inflammation leading to delayed milestones, and reduced lifespan in affected patients. There is currently no cure or treatment for LAMA2-CMD. Preclinical studies have demonstrated that mouse laminin-111 can serve as an effective protein replacement therapy in a mouse model of LAMA2-CMD.

**Methods:**

In this study, we generated a novel immunocompromised dy^W^ mouse model of LAMA2-CMD to study the role the immune system plays in muscle disease progression. We used this immune-deficient dy^W^ mouse model to test the therapeutic benefits of recombinant human laminin-111 and laminin-211 protein therapy on laminin-α2-deficient muscle disease progression.

**Results:**

We show that immunodeficient laminin-α2 null mice demonstrate subtle differences in muscle regeneration compared to immunocompetent animals during early disease stages but overall exhibit a comparable muscle disease progression. We found human laminin-111 and laminin-211 could serve as effective protein replacement strategies with mice showing improvements in muscle pathology and function. We observed that human laminin-111 and laminin-211 exhibit differences on satellite and myoblast cell populations and differentially affect muscle repair.

**Conclusions:**

This study describes the generation of a novel immunodeficient mouse model that allows investigation of the role the immune system plays in LAMA2-CMD. This model can be used to assess the therapeutic potential of heterologous therapies that would elicit an immune response. Using this model, we show that recombinant human laminin-111 can serve as effective protein replacement therapy for the treatment of LAMA2-CMD.

## Introduction

Laminin-α2-related congenital muscular dystrophy (LAMA2-CMD) also known as merosin-deficient congenital muscular dystrophy type 1A (MDC1A) is a severe genetic disease with an incidence estimated at 1–9/100,000 people and representing 10–30% of all congenital dystrophies [1–3]. LAMA2-CMD patients present with neonatal hypotonia, muscle wasting resulting in wheelchair confinement, and requiring respiratory support at a young age. There is currently no effective cure or treatment for LAMA2-CMD [4], and patients often die from respiratory insufficiency as early as during their first decade of life [5].

Although mutations can cause partial or reduced expression of LAMA2, mutations that result in a complete absence of the laminin-α2 protein chain cause the most severe muscle disease and clinical outcomes in patients. Laminin-α2 is a critical component of the laminin heterotrimer, which along with laminin-β1 or β2 and laminin-γ1 form the structural glycoproteins laminin-211 and laminin-221 in skeletal muscle. Laminin-211 and -221 polymerize with each other and interact with nidogen and collagen-IV to form the muscle basal lamina. Laminin-211 and laminin-221 bind to muscle cell surface through the α7β1 integrin and α-dystroglycan of the dystrophin glycoprotein complex via their globular C-terminal domains. This interaction anchors muscle cells to the basal lamina and regulates mechanotransduction and cell signaling [6, 7]. Loss of these laminin-211 and laminin-221 in LAMA2-CMD disrupts these molecular interactions and results in reduced muscle strength, failed muscle regeneration, inflammation, and fibrosis [1, 4, 5].

Severe inflammation is a hallmark of LAMA2-CMD and muscle biopsies from mouse and LAMA2-CMD patients exhibit immune cell infiltration especially during early stages of the disease. However, in contrast with other muscular dystrophies, inflammatory infiltrate is decreased during later stages of LAMA2-CMD muscle disease progression and its role in LAMA2-CMD muscle disease remains unclear [5, 8, 9].

In this study, to determine the role the immune response plays in LAMA2-CMD muscle disease, we produced an immunodeficient Lama2-null mouse on the dy^W^ strain background. This novel mouse line, which we designated NODScid dy^W^, lacks laminin-α2 and functional B and T-immune cells. This new model was compared to the immunocompetent dy^W^ mouse model to assess the impact of the loss of the immune response plays in LAMA2-CMD muscle disease. The immunodeficient NODScid dy^W^ mouse model showed reduced levels of basal regenerating myofibers compared to dy^W^ animals. There were no differences between immunodeficient and immunocompetent mice in terms of disease progression. These results indicate the immune response contributes to initial muscle disease in LAMA2-CMD, but that other non-immune-related mechanisms contribute to long-term muscle disease progression.

Laminin-111 is a form of laminin that is structurally and functionally similar to laminin-211 and 221 and has been shown to rescue mouse models of LAMA2-CMD [10–13]. We next determined the efficacy of recombinant human laminin-111 (HsLAM-111) and laminin-211 (HsLAM-211) protein therapies to prevent muscle disease progression using this immunodeficient dy^W^ mice model. Our results show that treatment with HsLAM-111 and HsLAM-211 improved muscle function and pathology, but results show HsLAM-111 and HsLAM-211 had different efficacies on muscle regeneration. Together these studies indicate a role for the adaptive immune response in LAMA2-CMD and support the idea of laminin protein replacement therapies as a treatment option for LAMA2-CMD.

## Results

### The NODScid dy^W^ mouse is immuno-deficient and lacks laminin-α2 protein

**Fig. 1:**
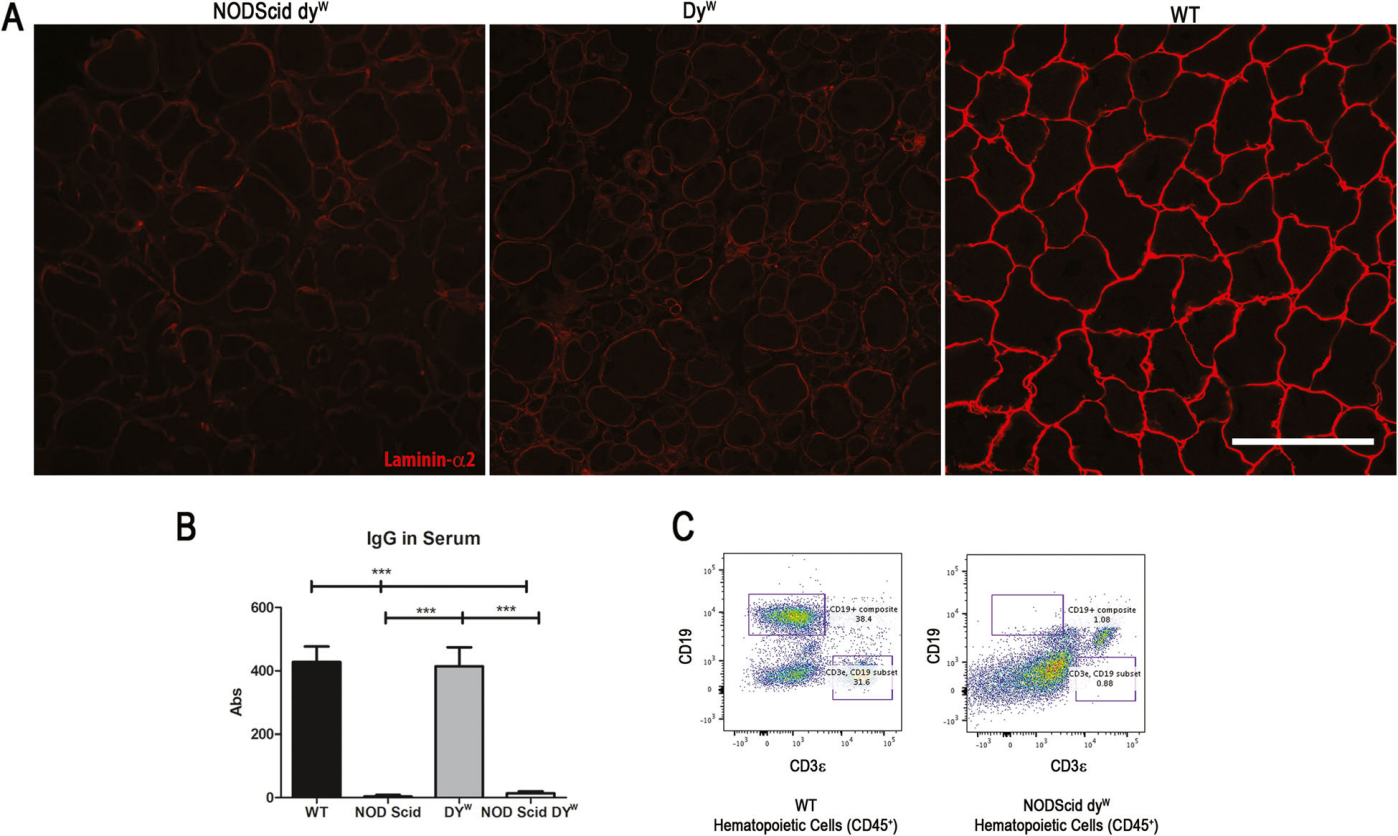
The NODScid dy^W^ mouse model of LAMA2-CMD shows downregulated levels of laminin-α2 protein chain and is immunodeficient. **a** Detection of laminin-α2 (red) in tibialis anterior (TA) sections of wild-type, dy^W^ internal control, dy^W^, and NODScid dy^W^. Scale bar 100 μm. **b** Relative levels of immunoglobulin in sera of wild-type, NOD Scid, dy^W^, and NODScid dy^W^ (*N* = 9; *p* value < 0.0001). **c** Fluorescence-activated cell sorting (FACS) gate analysis of hematopoietic cells (CD45^+^) from sera of wild-type and NODScid dy^W^ co-labeled with T cell marker (CD3ε^+^) and B cell marker (CD19^+^)

To produce an immunodeficient mouse model of LAMA2-CMD, NODScid mice were bred with dy^W+/−^ animals. Along with the test group NODScid dy^W^, we also generated wild-type, NODScid, and dy^W^ control groups. Muscles from wild-type, NODScid, dy^W^, and NODScid dy^W^ were harvested at 6 weeks of age and immunofluorescence used to detect the laminin-α2 chain. While strong signal for laminin-α2 immunofluorescence was observed in wild-type muscle, little signal was detected in dy^W^ and NODScid dy^W^ muscle (Fig. 1a). These results confirmed NODScid dy^W^ lacked laminin-α2 in skeletal muscle.

To determine if NODScid dy^W^ lacked an adaptive immune system, we next isolated serum from 6-week-old mice and performed an ELISA to detect serum immunoglobulin G (IgGs). Our results show that while wild-type and dy^W^ mice had high levels of IgG in serum, NODScid and the NODScid dy^W^ serum had no detectable IgGs (Fig. 1b). These results confirmed that NODScid dy^W^ animals lack functional B cells and are unable to produce immunoglobulin.

Next, we used fluorescence-activated-cell sorting (FACS) to quantify circulating levels of T and B cells in the blood. Hematopoietic cells (CD45^+^) from sera of wild-type and NODScid dy^W^ were co-labeled with T cell marker (CD3ε^+^) and B cell marker (CD19^+^) (Fig. 1c). Results showed that in wild-type, 31.6% of CD45^+^ cells were CD3ε^+^ and 38.4% were CD19^+^. In NODScid dy^W^, 0.88% were CD3ε^+^ and 1.08% were CD19^+^. These results show that NODScid dy^W^ mice lack functional T and B cells and therefore lack an adaptive immune response.

### Muscular dystrophy in NODScid dy^W^ is comparable to the dy^W^ mouse model of LAMA2-CMD

**Fig. 2:**
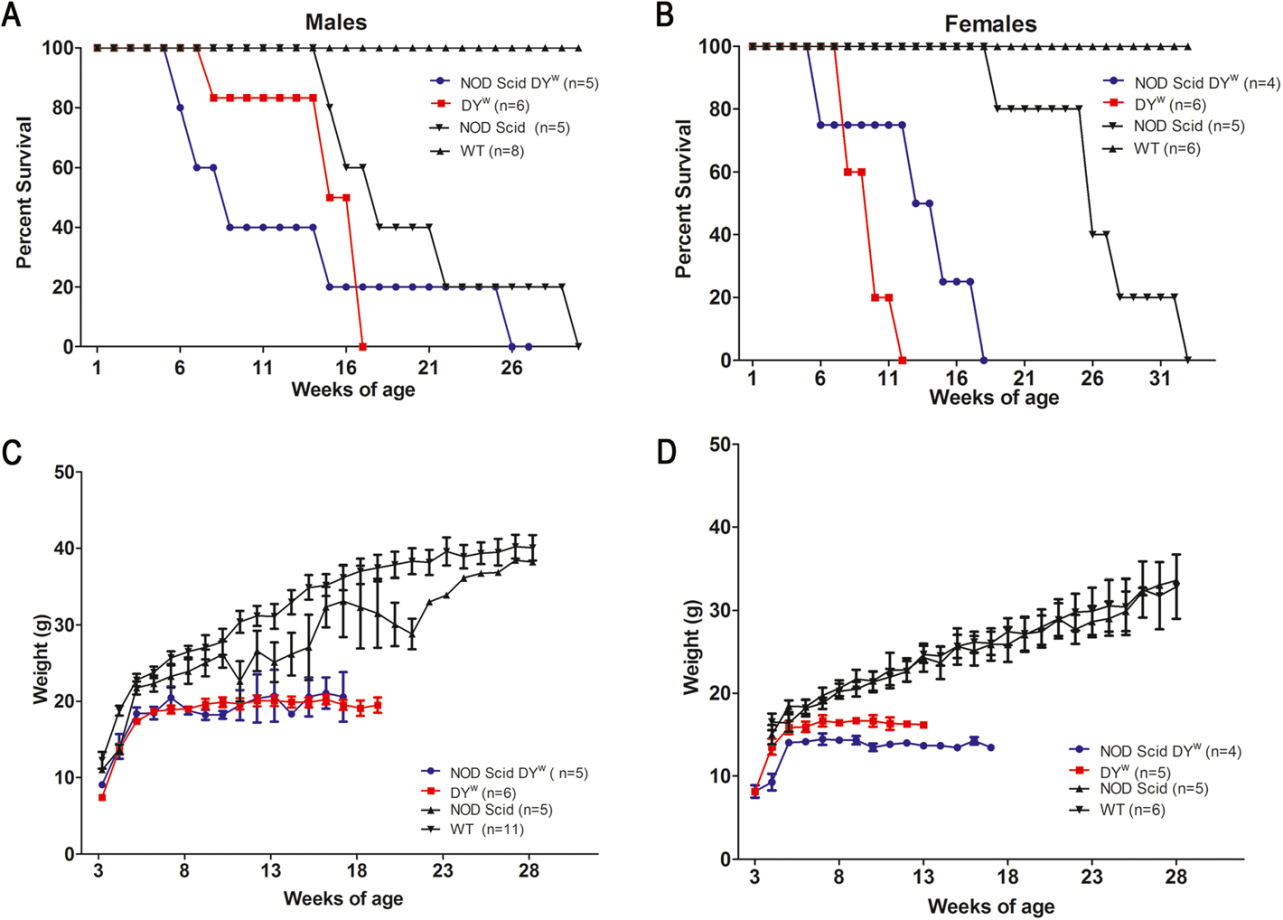
Survival and weight did not change in immunodeficient dy^W^ mice compared to immuno-competent mouse models of LAMA2-CMD. Survival study curves of male (**a**) and female (**b**) wild-type, NOD Scid, dy^W^, and NODScid dy^W^. **c** Weekly weights throughout the life span of male and female (**d**) wild-type, NOD Scid, dy^W^, and NODScid dy^W^

We next performed a survival study using wild-type, NODScid, dy^W^, and NODScid dy^W^ experimental groups. We observed that neither the female nor the male NODScid dy^W^ groups had a significant increase in lifespan compared to dy^W^ (Fig. 2a, b). Male and female NODScid showed reduced lifespan compared to wild-type mice, consistent with reports on radio-sensitivity-induced lymphomas [14]. Symptoms of lymphomas were not observed in the NODScid dy^W^ mouse. Weekly body mass showed no significant gender differences between NODScid dy^W^ and dy^W^ animals (Fig. 2c, d). These data indicate growth and survival were similar between immunocompetent and immunodeficient dy^W^ animals.

**Fig. 3:**
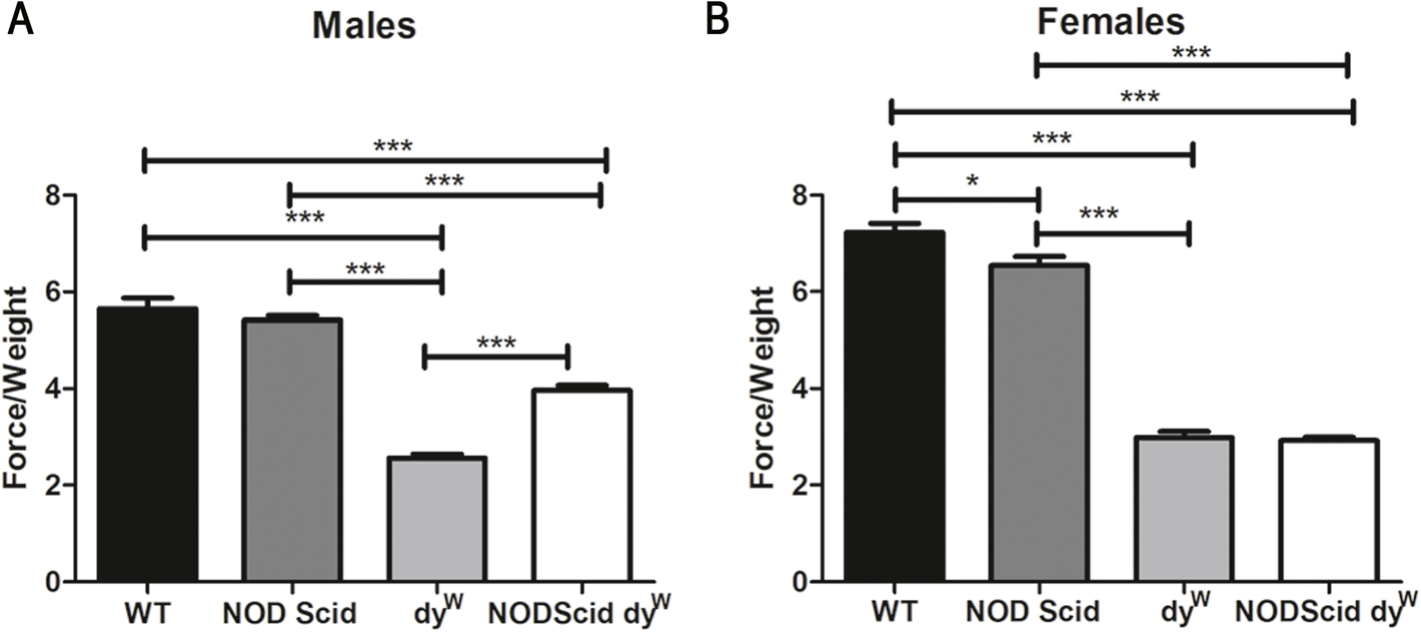
Grip strength is increased in immunodeficient male mice compared to immunocompetent LAMA2-CMD mouse models. Normalized force measurements of grip strength in wild-type, NOD Scid, dy^W^, and NODScid dy^W^**a** male (*N* = 4, 5, 7, 6, respectively; *p* value < 0.0001) and **b** female (*N* = 5; *p* value < 0.05*, < 0.0001***)

To determine if loss of the immune system affected muscle strength in dy^W^ mice, a forelimb grip-strength test was performed at 6 weeks of age and normalized to body weight as previously described [15]. As expected, 6-week-old wild-type and NODScid mice exhibited an average of 3-fold increase in muscle grip strength compared to dystrophic dy^W^ and NODScid dy^W^ groups. Interestingly, 6-week-old NODScid dy^W^ males showed a 1.4-fold increase in grip strength compared to dy^W^ males (Fig. 3a; *N* = 6 and 7, respectively, *p* value < 0.0001). In contrast, dy^W^ and NODScid dy^W^ females did not show any differences (Fig. 3b). These results suggest that the immune response in dystrophic muscle contributes to the lower grip strength observed in male dy^W^ animals.

To determine if the immune response in dystrophic muscle contributed to fibrosis observed in LAMA2-CMD, we used Sirius red to stain sections from TA muscles (Supplemental Figure 1A) and quantified levels of hydroxyproline as previously described [16] (Supplemental Figure 1B, C) in quadriceps of all groups. Sirius Red staining indicated more fibrosis in TA muscle sections from dy^W^ and NODScid dy^W^ mice compared to wild-type and NODScid muscle. This was confirmed and quantified using a hydroxyproline (HOP) assay. Wild-type and NODScid muscle had approximately 1.5-fold less HOP in their TA muscles compared to dystrophic dy^W^ and NODScid dy^W^ mice. There was no difference in HOP levels between males and females in the dy^W^ and NODScid dy^W^ experimental groups. These results indicate the immune response did not play a major role in the development of TA muscle fibrosis in 6-week-old dy^W^ mice.

To assess for the presence of other inflammatory cells, we used immunofluorescence to detect the three major myeloidal cell infiltrates: eosinophils, macrophages (CD11B) (Supplemental Figure 2), and neutrophils (LysC) (Supplemental Figure 3) associated with muscular dystrophy [8, 17]. Our results showed presence of innate immune infiltrates in NODScid dy^W^ and dy^W^ muscle, suggesting genetic ablation of the adaptive response through NODScid immune suppression did not affect the innate immune infiltration in these animals.

### Immunodeficient dy^W^ mice exhibit decreased muscle repair

**Fig. 4:**
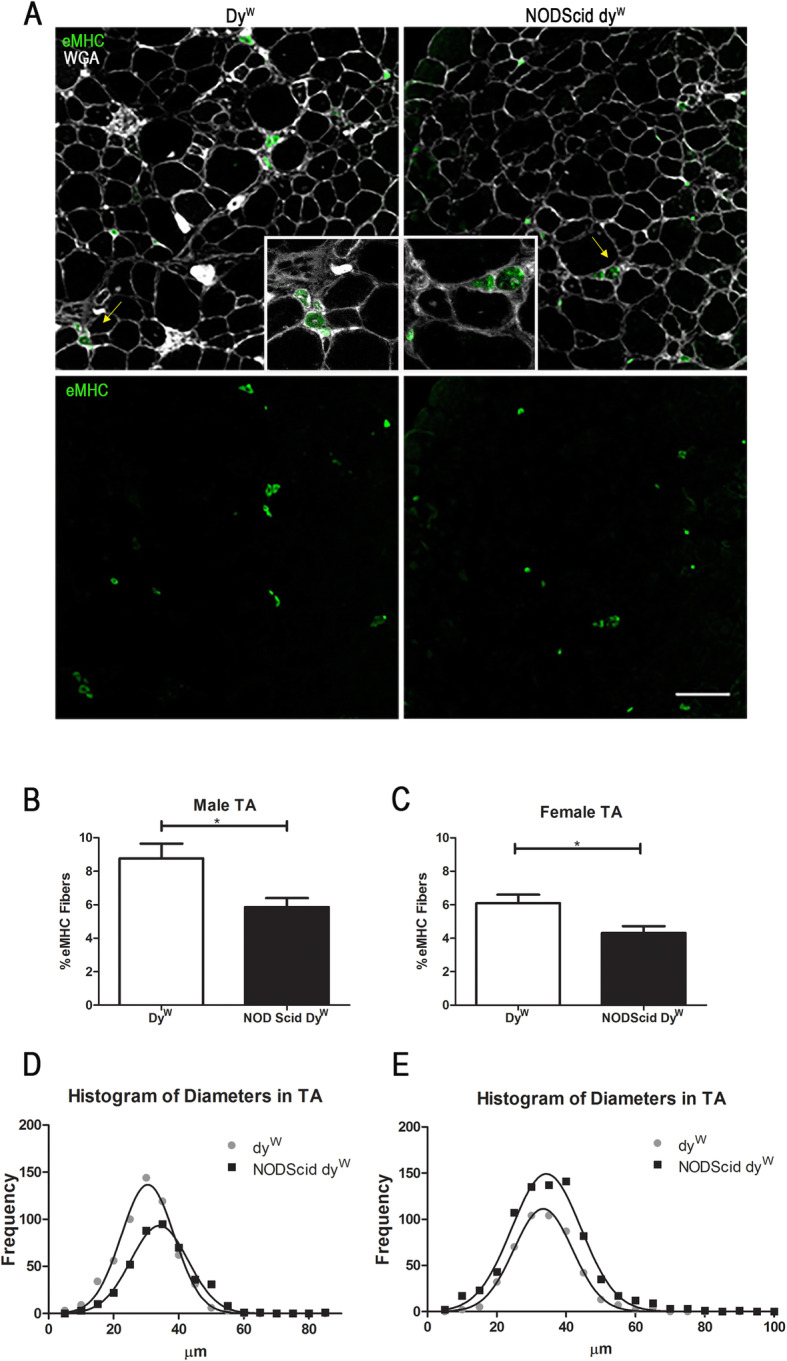
Basal regeneration is decreased in immunodeficient compared to immunocompetent mouse models of LAMA2-CMD. **a** Detection of embryonic Myosin Heavy Chain (eMHC) in TA sections of dy^W^ and NODScid dy^W^ mice. Quantification of eMHC-positive fibers in male (*N* = 7, 5, respectively; *p* value of 0.01; scale bar 100 μm) (**b**) and female (*N* = 5, 4, respectively; *p* value of 0.02). **c** dy^W^ and NODScid dy^W^ mice. Frequency histogram of minimum Feret’s diameters in TA muscle of male (*N* = 4, 5, respectively; *p* value < 0.0001) (**d**) and female (*N* = 5; *p* value < 0.0001) (**e**) dy^W^ and NODScid dy^W^ mice

Laminin-α2 deficiency leads to failed muscle regeneration and early apoptosis of regenerating myofibers [1, 18–20]. To assess differences in levels of ongoing regeneration in the NODScid dy^W^, we quantified embryonic myosin heavy chain (eMHC), a marker for muscle regeneration, in TA sections (Fig. 4a). When compared to dy^W^, we found that both male and female NODScid dy^W^ groups showed a decrease in eMHC-positive fibers: from 8.77 ± 0.81% in dy^W^ and 5.86 ± 0.535% in NODScid dy^W^ males (*N* = 7, 5, respectively, *p* value 0.01) and 6.09 ± 0.50% in dy^W^ and 4.31 ± 0.40% in NODScid dy^W^ females (*N* = 5, 4, respectively, *p* value 0.02) (Fig. 4b, c).

Feret minimal diameters were used to measure myofiber size. TA muscle from male mice showed a shift towards increased myofiber diameter, from a mean of 30.11 μm in dy^W^ to 34.81 μm in NODScid dy^W^ (*N* = 4, 5, respectively, *p* value < 0.0001). TA muscle from female mice, however, did not show a change in myofiber size with a mean of 34.00 μm in dy^W^ to 34.98 μm in NODScid dy^W^ (*N* = 5, *p* value 0.114) (Fig. 4d, e). This data suggests that NODScid dy^W^ muscle exhibits a lower level of basal muscle damage compared to dy^W^. This may indicate that suppression of adaptive immunity in laminin-α2-deficient skeletal muscle results in decreased muscle damage that results in muscle hypertrophy in male animals.

### Human laminin-111 and laminin-211 protein therapy increase muscle repair in NODScid dy^W^

Previous research has shown that treatment with natural Englebreth-Holm-Swam (EHS) murine sarcoma-derived laminin-111 enhances muscle regeneration and prevents myopathy of mouse models of LAMA2-CMD and Duchenne muscular dystrophy (DMD) [10, 11, 16, 21–23]. To test whether human laminin-111 has the same effect, we treated NODScid dy^W^ mice with HsLAM-111. We also used HsLAM-211 to investigate if it could completely substitute for the loss of laminin-α2 in LAMA2-CMD.

**Fig. 5:**
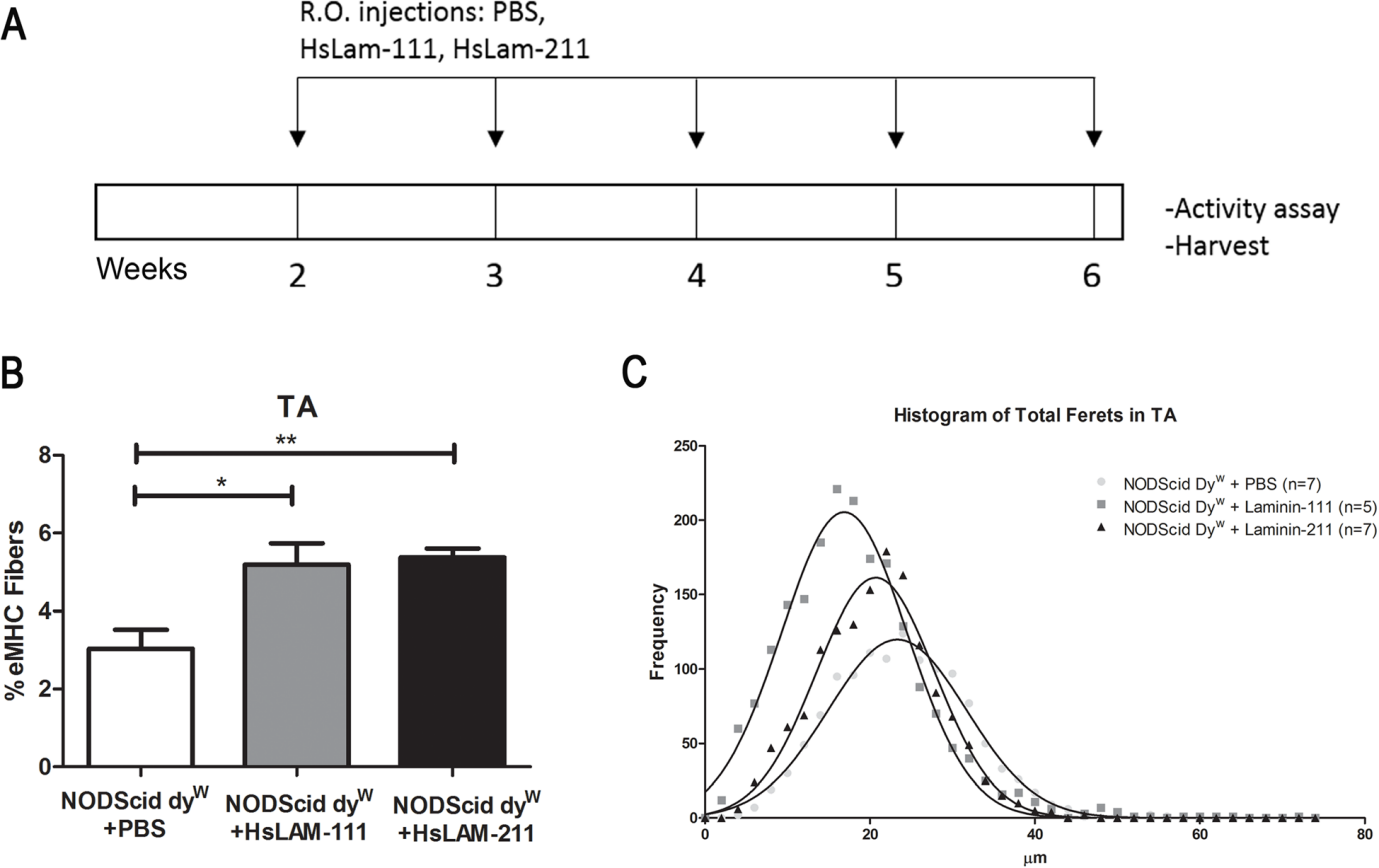
Treatment with human recombinant laminin-111 (HsLAM-111) and human recombinant laminin-211 (HsLAM-211) increased muscle regeneration in a mouse model of LAMA2-CMD. **a** NODScid dy^W^ mice were treated with weekly retro-orbital injections of 1 mg/kg HsLAM-111, HsLAM-211, or PBS from 2 weeks to 6 weeks of age. **b** Quantification of eMHC-positive fibers in PBS-, HsLAM-111-, and HsLAM-211-treated NODScid dy^W^ (*N* = 7, 5, 6, respectively; *p* value < 0.05*, 0.0049**). **c** Frequency histogram of minimum Feret’s diameters in TA muscle of PBS-, HsLAM-111-, and HsLAM-211-treated NODScid dy^W^ (*N* = 7, 5, 7, respectively; *p* value < 0.0001)

Female NODScid dy^W^ mice were treated from 2 to 6 weeks of age with weekly retro-orbital injections of 1 mg/kg HsLAM-111, HsLAM-211, or vehicle (Fig. 5a). This dose was 10-fold lower than previous studies [13, 16, 22] due to production availability of HsLAM-111 and HsLAM-211 (BioLamina, Sundyberg, Sweden) at 0.1 mg/ml compared to EHS laminin-111 (Thermo Fisher, Waltham MA) at 1 mg/ml. At 6 weeks of age, mice were humanely euthanized, and TA, gastrocnemius, quadriceps, and triceps were harvested.

TA muscle sections were tested for presence of laminin protein using immunofluorescence. Two antibodies directed against the carboxyl-terminal and rod domains of human laminin-α1 chain were used for immunofluorescence using muscle from vehicle and HsLAM-111 treated groups. Supplemental Fig. 4A shows positive immunofluorescence for both domains in HsLAM-111 compared to PBS-treated groups. Additionally, we used western analysis to show these antibodies are specific for the human isoform of laminin-111 (Supplemental Fig. 4B). We were unable to detect HsLAM-211 as antibodies against laminin-α2 were not specific for the human isoform and the dy^W^ mouse model expresses a low level of truncated laminin-α2 protein.

TA cryosections were subjected to immunofluorescence for eMHC-positive fibers. We found that treatment with both HsLAM-111 and HsLAM-211 resulted in a ~ 1.7-fold increase in levels of eMHC-positive fibers in laminin-treated muscle compared to vehicle treatment (Fig. 5b). Where PBS-treated mice showed 3.03 ± 0.48% eMHC-positive fibers, HsLAM-111 treatment resulted in a 5.192 ± 19% (*N* = 7, 5, respectively, *p* value < 0.05) and HsLAM-211 treatment in a 5.37 ± 0.23% (*N* = 7, 6; *p* value of 0.004).

We next measured myofiber size using Feret’s minimal diameters and observed a change in the mean from 23.68 μm in PBS-treated animals, 18.09 μm for HsLAM-111 treatment, and 20.76 μm in HsLAM-211 treatment (*N* = 7, 5, 7, respectively; *p* value < 0.0001). We observed a decrease in the standard deviations of myofiber size in laminin-treated muscles (SD) from 8.35 μm in PBS to 7.6 μm HsLAM-111 and 7.17 μm in HsLAM-211, indicating laminin treatment promoted a reduction in myofiber size variability (Fig. 5c). Centrally located nuclei (CLNs) fibers were also quantified as a measure of ongoing repair (data not shown) but showed no differences between vehicle and treatment groups.

Together, these data suggest that treatment with HsLAM-111 and HsLAM-211 increased muscle regeneration and reduced fiber diameter variability.

### Human laminin-111 and laminin-211 differentially affected myogenic cells in laminin-α2-deficient muscle

**Fig. 6:**
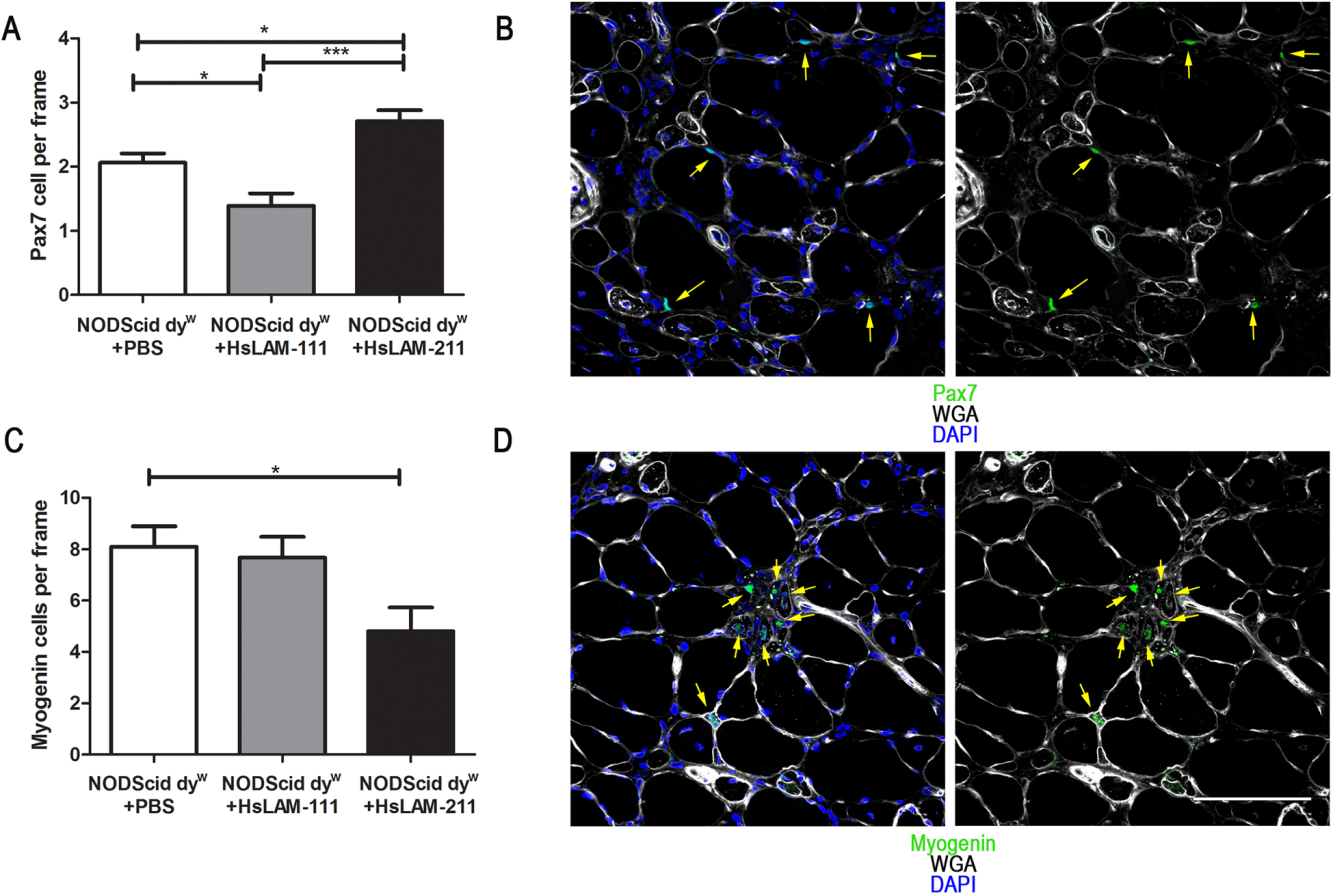
Treatment with HsLAM-111 and HsLAM-211 differentially affects satellite cells and myoblasts in skeletal muscle of LAMA2-CMD. **a** Quantification of Pax-7-positive satellite cells per frame in TA muscle sections of PBS-, HsLAM-111-, and HsLAM-211-treated NODScid dy^W^ (*N* = 8, 6, 6, respectively; *p* value < 0.05* < 0.0001***). Scale bar 100 μm. **b** Detection of Pax7-positive cells adjacent to fibers in TA sections of NODScid dy^W^ mice. **c** Quantification of myogenin-positive myoblasts per frame in TA muscle sections of PBS-, HsLAM-111-, and HsLAM-211-treated NODScid dy^W^ (*N* = 7; *p* value of 0.019). **d** Detection of myogenin-positive cells located interstitially in TA sections of NODScid dy^W^ mice

To test the effect of HsLAM-111 and HsLAM-211 on muscle repair, we next quantified satellite and myogenic cells in TA muscle. For satellite cells, we conducted immunofluorescence for the paired-box transcription factor 7 (Pax7) and counted the number of Pax7-positive cells located adjacent to the myofiber under the basal lamina (Fig. 6a). Treatment with HsLAM-111 resulted in a significant decrease in the number of satellite cells from 2.06 ± 0.14 cells per frame in PBS to 1.38 ± 0.19 Pax7-positive cells (*N* = 8, 6, respectively, *p* value < 0.05). In contrast, we observed a significant increase in satellite cells in muscle with HsLAM-211 treatment to 2.70 ± 0.17 satellite cells compared to HsLAM-111 or PBS treatments (*N* = 6, *p* value < 0.0001 and *p* < 0.05, respectively) (Fig. 6b).

To determine if recombinant human laminin treatment affected myogenic differentiation, we counted myogenin-positive cells (Fig. 6c). Our results showed no significant decrease of myogenin-positive cells from 8.1 ± 0.79 cells per area in PBS-treated animals to 7.56 ± 0.89 in HsLAM-111-treated mice. However, we did see a significant decrease to 4.78 ± 0.95 cells per frame in HsLAM-211-treated mice (*N* = 6, 7, respectively, *p* value of 0.01) (Fig. 6d).

Together, these results may suggest that laminin-111 and laminin-211 isoforms have an effect on muscle repair in laminin-α2 null muscle, while differentially promoting myogenic cell differentiation.

### Human laminin-111 treatment improves the activity of LAMA2-CMD mice

**Fig. 7:**
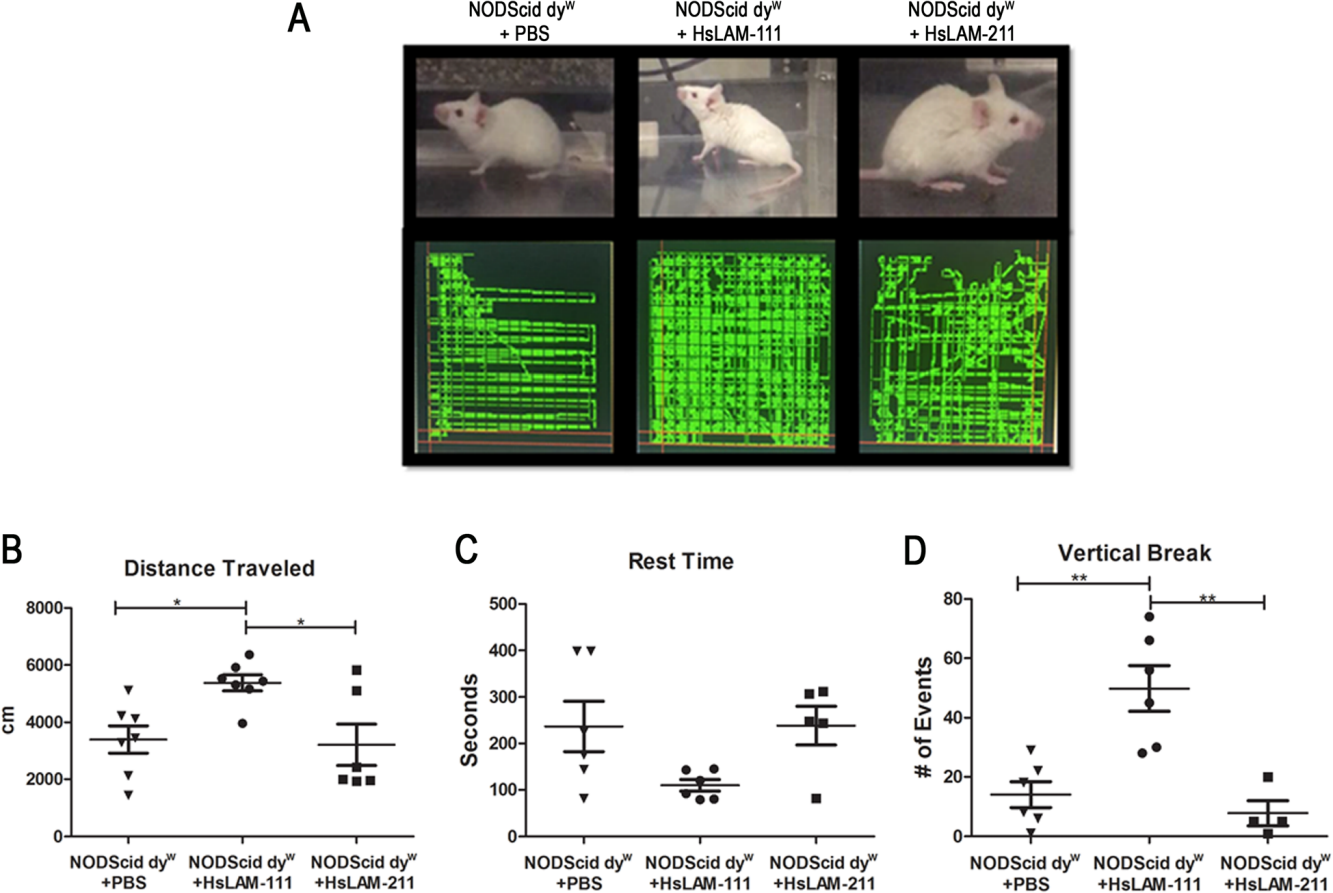
Treatment with HsLAM-111 improved mouse activity in LAMA2-CMD mouse model. **a** PBS-, HsLAM-111-, and HsLAM-211-treated NODScid dy^W^ mice pictures and trajectory detected using computer-controlled activity assay. Quantification of distance traveled **b**, resting time **c**, and vertical breaks **d** in PBS-, HsLAM-111-, and HsLAM-211-treated NODScid dy^W^ (*N* = 7, 7, 6, respectively; *p* value < 0.05*, < 0.005**)

Previous studies have shown that treatment with EHS-derived mouse laminin-111 improves muscle function in the dy^W^ mouse model. To test the human isoform of this biologic, NODScid dy^W^ mice were treated with HsLAM-111, HsLAM-211, or PBS for several weeks. Mice were then subjected to a computer-controlled activity assay as previously described [10] (Fig. 7a). Results showed a significant increase in distance traveled from a mean of 3394 ± 479 cm in PBS-treated mice to 5386 ± 281.7 cm in HsLAM-111 treatment (Fig. 7b) (*N* = 7, 7, respectively; *p* value < 0.05), but no increase in HsLAM-211-treated mice with 3211 ± 724.9 cm. Resting time showed no significant decrease from a mean of 236.8 ± 54.4 s in PBS to 109.8 ± 12.38 s in HsLAM-111 and 238.6 ± 41.66 in HsLAM-211 treatment groups (Fig. 7c). Finally, HsLAM-111-treated mice showed a significant increase in vertical breaks from 11.2 ± 9.5 in PBS to 50 ± 11.3 in HsLAM-111-treated animals, indicative of increased use of hindlimbs during the assay (Fig. 7d) (*N* = 7, 7; *p* value 0.0005). Grip strength was also performed with no significant differences between treatment groups (Supplemental Fig. 5) (*N* = 6). These data indicate treatment with recombinant human laminin-111 improves mobility of laminin-α2-deficient mice.

## Discussion

Mouse laminin-111 has previously shown to be an effective protein-based therapy in the dy^W^ mouse model of LAMA2-CMD [10, 13]. Although mouse and human laminin-111 have a high degree of homology, there are ~ 30% differences in the amino acid sequence in addition to differences in glycosylation patterns. For this reason, treatment using the recombinant HsLAM-111 is likely to generate an immune response in mice that could complicate interpretation of the efficacy of the human biologic. To investigate if recombinant human laminin-111 could prevent disease progression, we generated an immunodeficient dy^W^ mouse model of laminin-α2-related congenital muscular dystrophy (LAMA2-CMD). We demonstrate that this mouse model has disrupted expression of laminin-α2 protein and an ablated adaptive immune system. Histological and physiological characterization of this new mouse model shows severe muscle disease progression comparable to the established dy^W^ model of LAMA2-CMD.

The NOD Scid mouse has been shown to present multiple defects in innate and adaptive immunity and has been used extensively in xeno engraftment studies [24, 25]. Reduced natural killer (NK) cell activity, decrease in functionally mature macrophages, and ablation of functional lymphoid T and B cells are the main immuno-deficiencies reported in NODScid mice [14, 26]. The generation of a laminin-α2-deficient mouse that is also NODScid allows for a study of the role of the innate and adaptive immunity plays in LAMA2-CMD disease progression.

A case study of several laminin-α2-deficient patients reported high levels of T and B cell infiltration in skeletal muscle at an early age but decreased inflammatory infiltration at later stages of the disease [27]. This suggests that in contrast with other muscular dystrophies, the immune system in LAMA2-CMD may not play a major role in later stages of disease progression [8]. The immune response may, however, be important during neonatal stages and may exacerbate muscle disease progression. Our studies demonstrate there were no changes in the survival or weight of immunocompromised and immunocompetent dy^W^ animals. Lack of improved grip strength and hypertrophy in females as opposed to males is consistent with reports of more severe myopathy in females vs males in other LAMA2-CMD mouse models [28]. Another possible explanation may be sex-dependent differences in immune infiltration or timing between males and female during disease stages. The observed differences between male and female myopathy and inflammation in LAMA2-CMD remain to be further explored.

Loss of immune function did result in reduced muscle regeneration in NODScid dy^W^ mice as indicated with eMHC expression. The reduced levels of muscle regeneration and larger myofibers found in the NODScid dy^W^ mouse indicate of a role of an active immune response in promoting muscle regeneration in LAMA2-CMD.

Together, these results suggest the NODScid dy^W^ is a viable immunocompromised model for LAMA2-CMD, that while presenting reduced levels of basal muscle regeneration, disease progression is comparable to the established dy^W^ model and similar to LAMA2-CMD patients. This immunodeficient mouse model of LAMA2-CMD might be beneficial to investigate the role the immune system plays in LAMA2-CMD, or investigate the efficacy of human cell-based or human biologics for the treatment of LAMA2-CMD.

Using this novel mouse model of LAMA2-CMD, we next tested the efficacy of recombinant human laminin-111 and laminin-211 to act as protein substitution therapies and prevent muscle disease progression for LAMA2-CMD. Previous research has shown treatment with EHS murine laminin-111 can promote muscle regeneration and prevent muscular disease in mouse models of muscular dystrophy. To our knowledge, this is the first study to investigate the therapeutic potential of recombinant human laminin-111 and laminin-211 in a muscular dystrophy disease model.

Our results showed that HsLAM-111 and HsLAM-211 increased the regenerative capacity of muscle but that the mechanism of action of these laminin isoforms on satellite cells and myoblast wAS different. Laminin-111 promoted muscle repair at the expense of satellite cells, while laminin-211 preserved satellite cell populations in skeletal muscle. This could indicate that HsLAM-111 treatment induces satellite cell activation, which may explain the increase in eMHC-positive regenerative fibers observed in this treatment group. However, continuous long-term treatment with HsLAM-111 could deplete satellite cells. On the other hand, treatment with HsLAM-211 increased satellite cells and decreased myogenin-positive cells suggesting this laminin isoform could support the satellite cell niche in a way that preserves the satellite cell population. It is also possible that the local concentrations of laminin isoforms are critical for proliferation vs differentiation of myoblasts. Finally, we show that treatment with HsLAM-111 improved the activity of laminin-α2 null mice.

This short-term study of HsLAM-111 and HsLAM-211 in skeletal muscle provided preliminary data for the treatment of LAMA2-CMD. A long-term treatment using these biologicals is necessary to assess improvements in survival, immune infiltration, and fibrosis. Additionally, it is important to explore the long-term effect on satellite cell depletion and renewal, as well as the potential for the combination of HsLAM-111 and HsLAM-211 therapies.

To our knowledge, this is the first report using a laminin-α2-deficient mouse model to investigate the role that the immune system plays in LAMA2-CMD. In addition, this is the first report using an immunodeficient preclinical model to explore the short-term efficacy of recombinant human laminin-111 and laminin-211 on LAMA2-CMD disease progression. Limitations of this approach include producing enough purified HsLAM-111 to treat LAMA2-CMD patients, potential immune responses to glycosylation differences between recombinant and native human laminin isoforms, and the effects of long-term systemic treatment with HsLAM-111 protein.

## Materials and methods

### Generation of immunodeficient NODScid dy^W^ mice

All animal studies were performed under an approved animal protocol (#00404) reviewed by the Institutional Animal Care and Use Committee (IACUC) at the University of Nevada, Reno. To generate immunodeficient dy^W^ mice, female dy^W (+/−)^ mice were mated to male NOD.CB17-*Prkdc*^*SCID*^/J from Jackson Laboratories. Offspring were genotyped for LacZ gene inserted in dy^W^ and the NOD (non-obese diabetic) gene using PCR. The Scid gene was genotyped using qPCR using primers recommended by Jackson Laboratories. NOD^(+/−)^ Scid^(+/−)^ dy^W (+/−)^ were mated to generate breeding pairs NOD^(−/−)^ Scid^(−/−)^ dy^W (+/−)^ and NOD^(+/+)^ Scid^(+/+)^ dy^W (+/−)^. Matings of these mice generated wild-type, NODScid, dy^W^, and NODScid dy^W^ experimental groups.

### Survival study

Wild-type, NODScid, dy^W^, and NODScid dy^W^ were aged until they reached morbidity or death. Morbidity was described as loss of 10% weight from week to week or severe kyphosis combined with hind-limb myopathy as defined within the approved IACUC protocol.

### Generation and administration of recombinant laminins

HsLAM-111 and HsLAM-211 were purchased from BioLamina (Sundyberg, Sweden) where they are produced recombinantly at a stock concentration of 0.1 mg/ml. HsLAM-111 and HsLAM-211 dialyzed overnight against PBS to remove preservatives. Mice were anesthetized using isoflurane and administered each treatment group (HsLAM-111, HsLAM-211 or PBS) weekly via retro-orbital injection.

### Immunofluorescence

Muscles were harvested and embedded in 2:3 ratio of optimum cutting temperature (Fisher Scientific, Waltham, MA) and 30% sucrose medium prior to flash-freezing. Tissues were cryosectioned at 10-μm thick and stained with wheat germ agglutinin (WGA) (Vector Laboratories, Burlingame, CA, 1:00) for 10 min. Slides were prepared using Vectashield mounting media with DAPI stain (Vector Laboratories, Burlingame, CA). Immunostained sections were permeabilized using 0.2% Triton in PBS for 30 min before blocking, then primary antibodies anti-eMHC (Developmental Studies Hybridroma Bank (DSHB), Iowa City, IA, BF-45, 1:40), CD11B (Biolegend, San Diego, CA, 1:800) LysC (Biolegend, San Diego, CA, 1:800), Pax7 (DSHB, Iowa City, IA, Pax7, 1:20) and Myogenin (DSHB, Iowa City, IA, F5D, 1:20) were incubated overnight. Rabbit polyclonal antibodies against human laminin-α1C-terminal and rod domains (Prothelia, Inc., Milford, MA) were used at a concentration of 1:20 without permeabilization. All primary antibodies were followed by secondary antibody incubation for 1 h using FITC-anti-mouse or FITC-anti-rabbit antibodies (Jackson Laboratories, Sacramento, CA, 1:200), except for pre-labeled FITC-CD11b and FITC-LysC, and lastly incubated for 10 min WGA stain (WGA-647 1:100). Slides were imaged using the Olympus Fluoview FV 1000 laser confocal microscope and analyzed using Image J-win32 software. Whole muscle cross-sections were imaged and used for quantification of fiber diameters and eMHC. We obtained 5 to 10 images at × 40 magnification to quantify Pax7- and myogenin-positive cells. To measure fibrosis TA slides were stained with Sirius Red (Sigma-Aldrich, St. Louis, MI) as previously described (Van Ry, 2014). Images were captured using Axiovision 4.8 software.

### Immunoblotting

At total of 1 mg of HsLam-111, HsLam-211, and EHS laminin-111 (Thermo Scientific, Waltham, MA) proteins were separated in a NuPAGE 4 to 12% Bis-Tris gel (Thermo Scientific, Waltham, MA) and transferred onto a Nitrocellulose membrane. Laminin-111 C-terminal and rod-terminal domains were detected using home-made antibodies at a 1:100 concentration incubated overnight followed by 1-h incubation with secondary antibody Alexa Fluor680 conjugated anti-rabbit (Invitrogen, Carlsbad, CA). LI-COR imaging system was used to detect and image protein bands.

### IgG detection

An enzyme-linked immunoabsorbent assay (ELISA) was used to measure the relative levels of immunoglobulin (IgG) in mouse sera. Mouse sera were used to coat an Immulon 1B (Thermo Scientific, Waltham, MA) plate in triplicates overnight at 4C. After three 0.1%SDS washes, samples were incubated with mouse anti-IgG antibody at 1:200 dilution in 1% BSA overnight at 4C. After three more washes, samples were incubated with secondary antibody FITC-anti-mouse (Jax labs, Bar Harbor, ME). After the final wash, plate was read using Victor photospectrometer at 500 nm.

### Fluorescence-activated cell sorting (FACS)

Blood samples were drawn from 6-week-old mice into EDTA K3 coated tubes. One million cells were diluted with pre-labeled primary antibodies CD45 (1:800), CD19 (1:800), and CD3ε (1:100) from BD Biosciences (San Jose, CA) and incubated for 30 min in the dark at room temperature. Cells were lysed using FACS lysis solution protocol (BD Biosciences Cat.no 349202) and washed in 0.5% fetal bovine serum in PBS. Cells were quantified using the BD LSR II SORP cell analyzer and data was analyzed using FlowJo software.

### Statistics

GraphPad Prism software was used for statistical calculations. Student’s *t* test was used to compare means between two groups and one-way ANOVA was used to compare means between three or more groups. All ANOVA calculations were followed by Bonferroni post-test. Means of experimental groups were considered statistically significant when *p* < 0.05.

## Supplementary information

**Additional file 1: **Supplemental Figure 1. Fibrosis is not significantly changed between immuno deficient and immuno competent LAMA2-CMD muscle. (A) Detection of sirius red stain in TA sections of wild type, NOD Scid, dyW and NODScid dyW mice. Quantification of hydroxyproline in quadriceps of male (*N* = 4, 6, 8, 5 respectively; p value < 0.05*, 0.004**) (B) and female male (*N* = 5, 4, 5, 6 respectively; p value < 0.05*, 0.0014**) (C) wild type, NOD Scid, dyW and NODScid dyW mice. Supplemental Figure 2. Macrophages and eosinophils are not severely changed in immuno deficient compared to immuno competent LAMA2-CMD muscle. (A) Detection of CD11B positive cells in TA sections of NODScid dyW, dyW and wild type. Scale bar 100 μm. Supplemental Figure 3. Neutrophils are not severely changed in immuno deficient compared to immuno competent LAMA2-CMD muscle. (A) Detection of LysC positive cells in TA sections of NODScid dyW, dyW and wild type. Scale bar 100 μm. Supplemental Fig 4. Immunofluorescence shows positive staining of human Laminin-111 in TA of immunocompromised LAMA2-CMD mice treated with human recombinant Laminin-111. HsLam-111-treated NODScid DyW show positive staining against human Laminin-111 C-terminal domain (A) and rod terminal domain (B) compared to PBS-treated mice. Antibodies are specific for human Laminin-111 compared to mouse and 211 isoforms. Western blot of 1 μg of mouse Laminin-111, HsLam-111 and HsLam-211 protein probed against α-human Laminin-111 (C) rod-domain and (D) C-terminal domain. Supplemental Fig 5. Treatment with human Laminin-111 or 211 does not significantly change grip strength in an immunocompromised mouse model of LAMA2-CMD. (A) HsLam-111 and HsLam-211-treated NSDyW mice did not show a significant increase in grip strength when compared to PBS-treated group (*N* = 6).

## Data Availability

Most of the data generated and analyzed during this study are included in the manuscript or supplemental data. Any data not included will be made available from the corresponding author upon request.

## Abbreviations

ANOVA: Analysis of variance
B cells: Bursa of Fabricius cells
CD: Cluster of differentiation
C-terminal: Carboxyl-terminal domain
DSHB: Developmental Studies Hybridroma Bank
DMD: Duchenne muscular dystrophy
EHS: Englebreth-Holm-Swam
ELISA: Enzyme-linked immunoabsorbent assay
eMHC: Myosin heavy chain
FACS: Fluorescence-activated cell sorting
FITC: Fluorescein isothiocyanate
HOP: Hydroxyproline
HsLAM-111: Laminin-111
HsLAM-211: Laminin-211
IACUC: Institutional Animal Care and Use Committee
LAMA2-CMD: Laminin-α2-related congenital muscular dystrophy
IgG: Immunoglobulin G
MDC1A: Merosin-deficient congenital muscular dystrophy type 1A
NOD: Non-obese diabetic
Pax7: Paired-box transcription factor 7
PBS: Phosphate-buffered saline
PCR: Polymerase chain reaction
qPCR: Quantitative polymerase chain reaction
*p* value: Probability value
Scid: Severe combined immuno-deficiency
TA: Tibialis anterior
T cells: Thymus lymphocyte cells
WGA: Wheat germ agglutinin

## References

[1] Mohassel P, Reghan Foley A, Bönnemann CG. Extracellular matrix-driven congenital muscular dystrophies [Internet]. Matrix Biol. 2018 [cited 2019 Apr 25]. p. 188–204. Available from. 10.1016/j.matbio.2018.06.005. 10.1016/j.matbio.2018.06.005 29933045

[2] Sframeli M, Sarkozy A, Bertoli M, Astrea G, Hudson J, Scoto M (2017). Congenital muscular dystrophies in the UK population: clinical and molecular spectrum of a large cohort diagnosed over a 12-year period. Neuromuscul Disord..

[3] N. D, M. T. Neuromuscular disorders in childhood: a descriptive epidemiological study from western Sweden. Neuromuscul Disord [Internet]. 2000;10:1–9. Available from: http://www.embase.com/search/results?subaction = viewrecord&from = export&id = L30006857%0Ahttp://10.1016/S0960-8966(99)00055-3. 10677857

[4] Nguyen Q, Lim KRQ, Yokota T. Current understanding and treatment of cardiac and skeletal muscle pathology in laminin-α2 chain-deficient congenital muscular dystrophy. Appl Clin Genet [Internet]. 2019 [cited 2019 Aug 22];Volume 12:113–30. Available from: 10.2147/TACG.S187481. 10.2147/TACG.S187481 PMC6618038 31308722

[5] Konkay K, Kannan M, Lingappa L, Uppin M, Challa S (2016). Congenital muscular dystrophy with inflammation: diagnostic considerations. Ann Indian Acad Neurol..

[6] Patton BL. Laminins of the neuromuscular system [Internet]. Microsc. Res. Tech. 2000 [cited 2019 Mar 16]. p. 247–61. Available from: http://www.ncbi.nlm.nih.gov/pubmed/11054875. 10.1002/1097-0029(20001101)51:3<247::AID-JEMT5>3.0.CO;2-Z 11054875

[7] Patton BL, Miner JH, Chiu AY, Sanes JR. Distribution and function of laminins in the neuromuscular system of developing, adult, and mutant mice. J Cell Biol [Internet]. 1997 [cited 2019 Mar 16];139:1507–21. Available from: http://www.jcb.org. 10.1083/jcb.139.6.1507 PMC2132624 9396756

[8] Tidball JG, Welc SS, Wehling-Henricks M. Immunobiology of inherited muscular dystrophies. Compr Physiol [Internet]. 2018 [cited 2019 May 8];8:1313–56. Available from: https://onlinelibrary-wiley-com.unr.idm.oclc.org/doi/pdf/10.1002/cphy.c170052. 10.1002/cphy.c170052 PMC7769418 30215857

[9] Pegoraro E, Cianno B Di, Hoffman EP, Mancias P, Swerdlow SH, Raikow RB, et al. Congenital muscular dystrophy with primary laminin α2 (merosin) deficiency presenting as inflammatory myopathy. Ann Neurol [Internet]. 2005 [cited 2019 Jul 9];40:782–91. Available from: https://onlinelibrary-wiley-com.unr.idm.oclc.org/doi/pdf/10.1002/ana.410400515. 10.1002/ana.410400515 8957020

[10] Rooney JE, Knapp JR, Hodges BL, Wuebbles RD, Burkin DJ. Laminin-111 protein therapy reduces muscle pathology and improves viability of a mouse model of merosin-deficient congenital muscular dystrophy. Am J Pathol [Internet]. Elsevier Inc.; 2012;180:1593–602. Available from: 10.1016/j.ajpath.2011.12.019. 10.1016/j.ajpath.2011.12.019 PMC3349899 22322301

[11] Gawlik KI, Harandi VM, Cheong RY, Petersén Å, Durbeej M. Laminin α1 reduces muscular dystrophy in dy2Jmice. Matrix Biol [Internet]. 2018 [cited 2018 Mar 26]; Available from: https://ac.els-cdn.com/S0945053X1830043X/1-s2.0-S0945053X1830043X-main.pdf?_tid = 2812fba9-3b9b-41d3-a452-ac0615da372b&acdnat = 1522110251_a4d296cbed8c6f6ced9d0014538bd588. 10.1016/j.matbio.2018.02.024 29544677

[12] Gawlik KI, Mayer U, Blomberg K, Sonnenberg A, Ekblom P, Durbeej M. Laminin α1 chain mediated reduction of laminin α2 chain deficient muscular dystrophy involves integrin α7β1 and dystroglycan. FEBS Lett [Internet]. 2006 [cited 2019 Aug 15];580:1759–65. Available from: https://febs-onlinelibrary-wiley-com.unr.idm.oclc.org/doi/pdf/10.1016/j.febslet.2006.02.027. 10.1016/j.febslet.2006.02.027 16504180

[13] van Ry PM, Minogue P, Hodges BL, Burkin DJ. Laminin-111 improves muscle repair in a mouse model of merosin-deficient congenital muscular dystrophy. Hum Mol Genet [Internet]. Oxford University Press; 2014 [cited 2016 Sep 6];23:383–96. Available from: http://www.hmg.oxfordjournals.org/cgi/doi/10.1093/hmg/ddt428. 10.1093/hmg/ddt428 PMC3869356 24009313

[14] Shultz LD, Schweitzer PA, Christianson SW, Gott B, Schweitzer IB, Tennent B, et al. Multiple defects in innate and adaptive immunologic function in NOD/LtSz-scid mice. J Immunol [Internet]. 1995 [cited 2019 Apr 7];154:180–91. Available from: http://www.jimmunol.org/content/154/1/180. 7995938

[15] Welser J V, Rooney JE, Cohen NC, Gurpur PB, Singer CA, Evans RA, et al. Myotendinous junction defects and reduced force transmission in mice that lack α7 integrin and utrophin. Am J Pathol [Internet]. 2009 [cited 2019 Jul 11];175:1545–54. Available from: https://www.ncbi.nlm.nih.gov/pmc/articles/PMC2751551/pdf/JPATH175001545.pdf. 10.2353/ajpath.2009.090052 PMC2751551 19729483

[16] Barraza-Flores P, Fontelonga TM, Wuebbles RD, Hermann HJ, Nunes AM, Kornegay JN, et al. Laminin-111 protein therapy enhances muscle regeneration and repair in the GRMD dog model of Duchenne muscular dystrophy. Hum Mol Genet [Internet]. 2019 [cited 2019 Jul 11]; Available from: https://academic.oup.com/hmg/advance-article-abstract/doi/10.1093/hmg/ddz086/5479258. 10.1093/hmg/ddz086 PMC6687953 31179490

[17] Wosczyna MN, Rando TA (2018). A muscle stem cell support group: coordinated cellular responses in muscle regeneration. Dev Cell..

[18] Kuang W, Xu H, Vilquin JT, Engvall E. Activation of the lama2 gene in muscle regeneration: abortive regeneration in laminin alpha2-deficiency. Lab Invest [Internet]. 1999 [cited 2019 Jul 17];79:1601–13. Available from: http://www.ncbi.nlm.nih.gov/pubmed/10616210. 10616210

[19] Yurchenco PD, McKee KK, Reinhard JR, Rüegg MA. Laminin-deficient muscular dystrophy: molecular pathogenesis and structural repair strategies [Internet]. Matrix Biol. 2018 [cited 2019 Feb 4]. p. 174–87. Available from: 10.1016/j.matbio.2017.11.009. 10.1016/j.matbio.2017.11.009 PMC5971131 29191403

[20] North KN, Specht LA, Sethi RK, Shapiro F, Beggs AH. Congenital muscular dystrophy associated with merosin deficiency the congenital muscular dystrophies are a heterogeneous group of muscle diseases characterized by early-onset weak-ness, hypotonia, delayed motor milestones, and a high inci-dence of severe [Internet]. J Child Neurol. 1996. Available from: https://journals-sagepub-com.unr.idm.oclc.org/doi/pdf/10.1177/088307389601100406. 10.1177/088307389601100406 8807418

[21] Gawlik KI, Li J-Y, Petersén A, Durbeej M. Laminin alpha1 chain improves laminin alpha2 chain deficient peripheral neuropathy. Hum Mol Genet [Internet]. 2006;15:2690–700. Available from: http://www.ncbi.nlm.nih.gov/pubmed/16893907. 10.1093/hmg/ddl201 16893907

[22] Rooney JE, Gurpur PB, Burkin DJ. Laminin-111 protein therapy prevents muscle disease in the mdx mouse model for Duchenne muscular dystrophy. Proc Natl Acad Sci U S A. United States; 2009. p. 7991–6. 10.1073/pnas.0811599106 PMC2683113 19416897

[23] Riederer I, Bonomo AC, Mouly V, Savino W. Laminin therapy for the promotion of muscle regeneration. FEBS Lett [Internet]. Federation of European Biochemical Societies; 2015 [cited 2019 Jun 14];589:3449–53. Available from: 10.1016/j.febslet.2015.10.004. 10.1016/j.febslet.2015.10.004 26459029

[24] Shultz LD, Ishikawa F, Greiner DL. Humanized mice in translational biomedical research [Internet]. Nat. Rev. Immunol. 2007 [cited 2019 Apr 10]. p. 118–30. Available from: www.nature.com/reviews/immunol. 10.1038/nri2017 17259968

[25] Mice CB, Greiner DL, Shultz LD, Yates J, Appel MC, Perdrizet G, et al. Improved engraftment of human spleen cells in NODILtSz-scid/scid mice as compared with C.B-1 7-scid/scid Mice. Am J Pathol [Internet]. 1995 [cited 2019 Jul 9];146:888–902. Available from: https://www.ncbi.nlm.nih.gov/pmc/articles/PMC1869266/pdf/amjpathol00052-0110.pdf. PMC1869266 7717456

[26] Prochazka M, Gaskins HR, Shultz LD, Leiter EH. The nonobese diabetic scid mouse: Model for spontaneous thymomagenesis associated with immunodeficiency (severe combined immunodeficiency mutation). Immunology [Internet]. 1992;89:3290–4. Available from: https://www.ncbi.nlm.nih.gov/pmc/articles/PMC48852/pdf/pnas01082-0136.pdf. 10.1073/pnas.89.8.3290 PMC48852 1373493

[27] Pegoraro E, Mancias P, Swerdlow SH, Raikow RB, Garcia C, Marks H, et al. Congenital muscular dystrophy with primary laminin-a2 (Merosin) deficiency presenting as inflammatory myopathy. Am Neurol Assoc [Internet]. 1996 [cited 2019 May 13];782–91. Available from: https://onlinelibrary-wiley-com.unr.idm.oclc.org/doi/pdf/10.1002/ana.410400515. 10.1002/ana.410400515 8957020

[28] Fontes-Oliveira CC, M. Soares Oliveira B, Körner Z, M. Harandi V, Durbeej M. Effects of metformin on congenital muscular dystrophy type 1A disease progression in mice: a gender impact study. Sci Rep [Internet]. 2018 [cited 2019 Apr 24];8. Available from: www.nature.com/scientificreports/. 10.1038/s41598-018-34362-2 PMC6214987 30389963

