# Supplementary material for: Human laminin-111 and laminin-211 protein therapy prevents muscle disease progression in an immunodeficient mouse model of LAMA2-CMD
Source: Skelet Muscle. 2020 Jun 4;10:18. doi: 10.1186/s13395-020-00235-4 (PMC7271547; doi:10.1186/s13395-020-00235-4)
Supplement: Supplementary file 1 — Additional file 1: Supplemental Figure 1. Fibrosis is not significantly changed between immuno deficient and immuno competent LAMA2-CMD muscle. (A) Detection of sirius red stain in TA sections of wild type, NOD Scid, dyW and NODScid dyW mice. Quantification of hydroxyproline in quadriceps of male (N = 4, 6, 8, 5 respectively; p value < 0.05*, 0.004**) (B) and female male (N = 5, 4, 5, 6 respectively; p value < 0.05*, 0.0014**) (C) wild type, NOD Scid, dyW and NODScid dyW mice. Supplemental Figure 2. Macrophages and eosinophils are not severely changed in immuno deficient compared to immuno competent LAMA2-CMD muscle. (A) Detection of CD11B positive cells in TA sections of NODScid dyW, dyW and wild type. Scale bar 100 μm. Supplemental Figure 3. Neutrophils are not severely changed in immuno deficient compared to immuno competent LAMA2-CMD muscle. (A) Detection of LysC positive cells in TA sections of NODScid dyW, dyW and wild type. Scale bar 100 μm. Supplemental Fig 4. Immunofluorescence shows positive staining of human Laminin-111 in TA of immunocompromised LAMA2-CMD mice treated with human recombinant Laminin-111. HsLam-111-treated NODScid DyW show positive staining against human Laminin-111 C-terminal domain (A) and rod terminal domain (B) compared to PBS-treated mice. Antibodies are specific for human Laminin-111 compared to mouse and 211 isoforms. Western blot of 1 μg of mouse Laminin-111, HsLam-111 and HsLam-211 protein probed against α-human Laminin-111 (C) rod-domain and (D) C-terminal domain. Supplemental Fig 5. Treatment with human Laminin-111 or 211 does not significantly change grip strength in an immunocompromised mouse model of LAMA2-CMD. (A) HsLam-111 and HsLam-211-treated NSDyW mice did not show a significant increase in grip strength when compared to PBS-treated group (N = 6). [file 13395_2020_235_MOESM1_ESM.docx]

**Supplemental Figures**


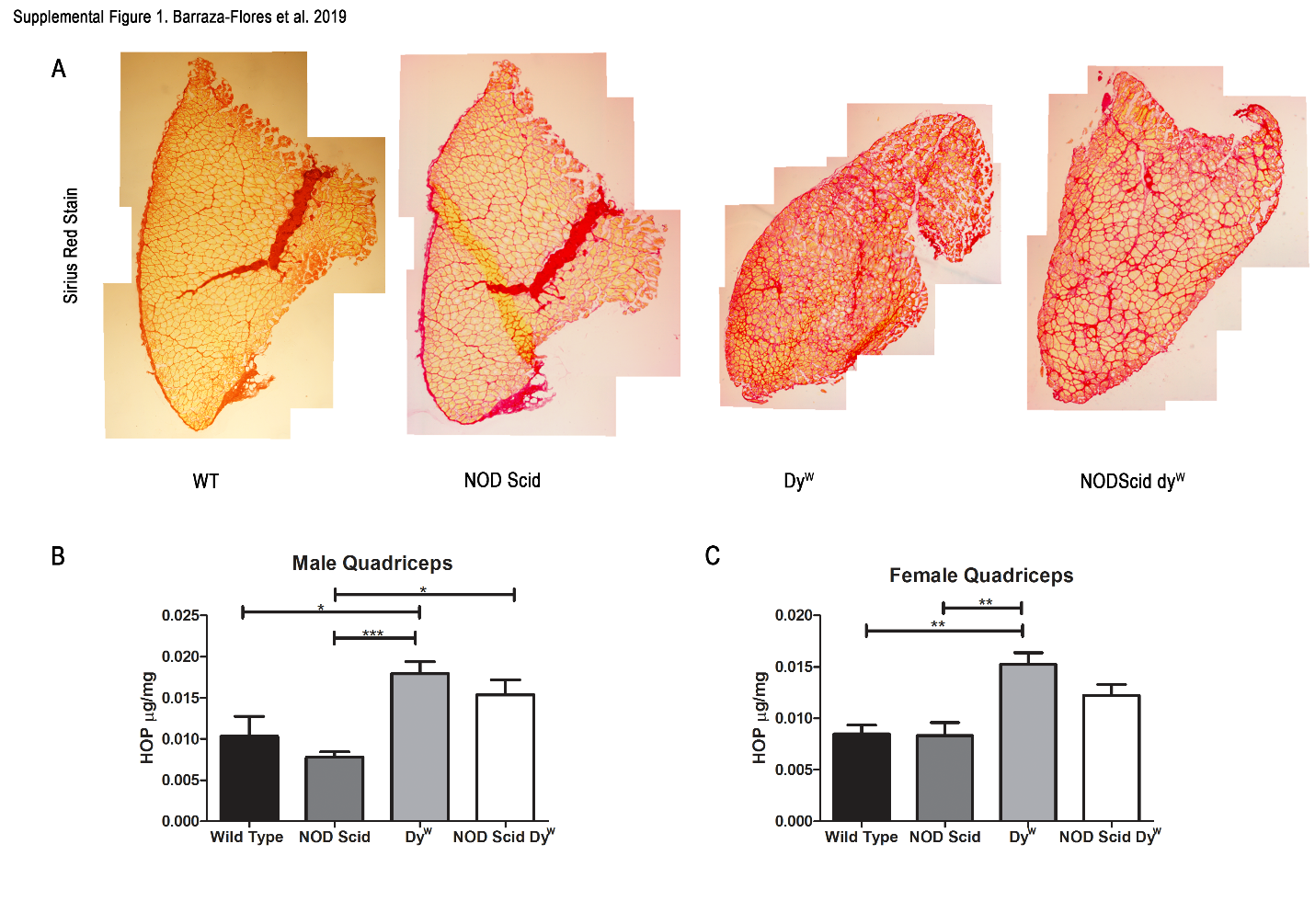


Supplemental Figure 1. Fibrosis is not significantly changed between immuno deficient and immuno competent LAMA2-CMD muscle. **(A)** Detection of sirius red stain in TA sections of wild type, NOD Scid, dy^W^ and NODScid dy^W^ mice. Quantification of hydroxyproline in quadriceps of male (N=4, 6, 8, 5 respectively; p-value <0.05*, 0.004**) **(B)** and female male (N=5, 4, 5, 6 respectively; p-value <0.05*, 0.0014**) **(C)** wild type, NOD Scid, dy^W^ and NODScid dy^W^ mice.


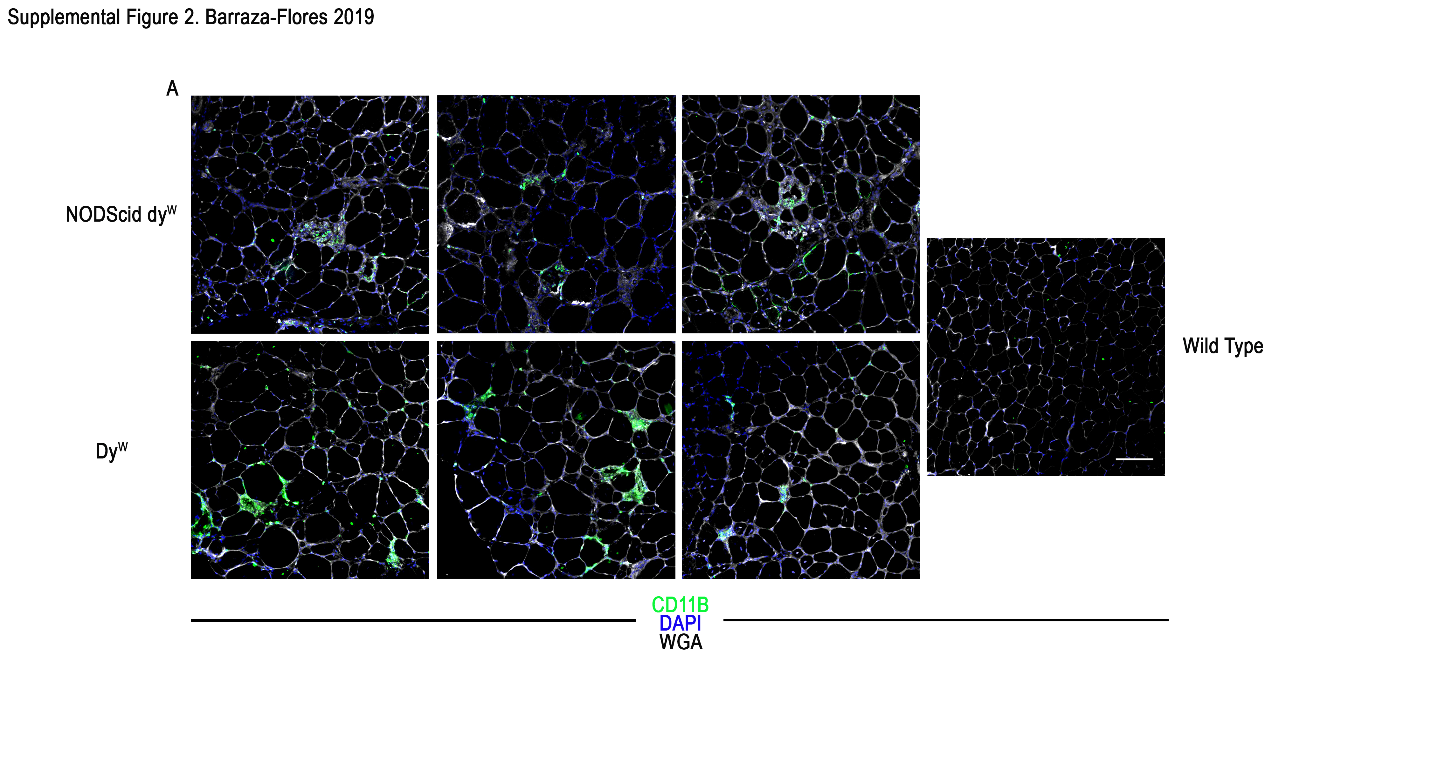


Supplemental Figure 2. Macrophages and eosinophils are not severely changed in immuno deficient compared to immuno competent LAMA2-CMD muscle. **(A)** Detection of CD11B positive cells in TA sections of NODScid dyW, dyW and wild type. Scale bar 100 µm.


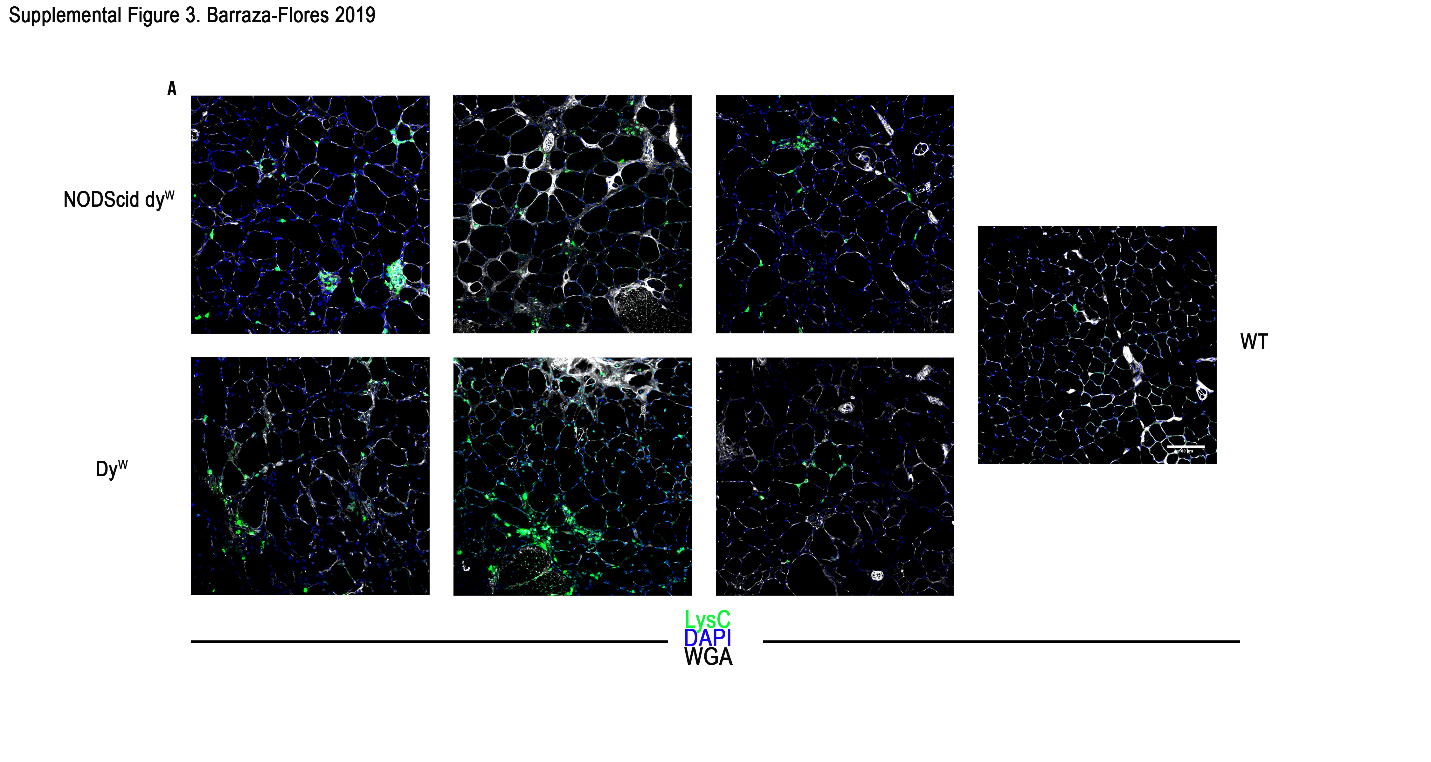


Supplemental Figure 3. Neutrophils are not severely changed in immuno deficient compared to immuno competent LAMA2-CMD muscle. **(A)** Detection of LysC positive cells in TA sections of NODScid dyW, dyW and wild type. Scale bar 100 µm.

| 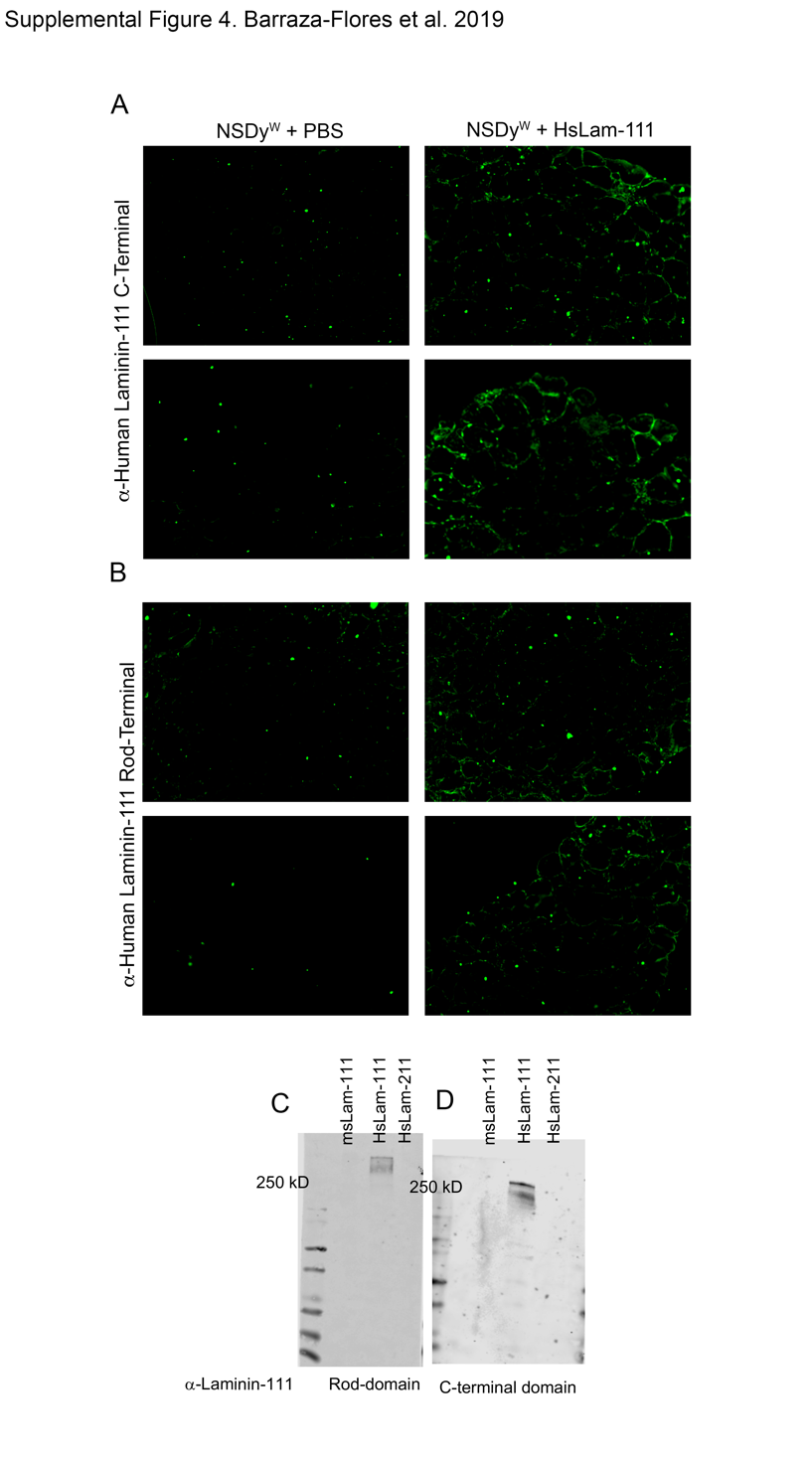 |
| --- |

Supplemental Fig 4. Immunofluorescence shows positive staining of human Laminin-111 in TA of immunocompromised LAMA2-CMD mice treated with human recombinant Laminin-111. HsLam-111 treated NODScid Dy^W^ show positive staining against human Laminin-111 C-terminal domain **(A)** and rod terminal domain **(B)** compared to PBS treated mice. Antibodies are specific for human Laminin-111 compared to mouse and 211 isoforms. Western blot of 1μg of mouse Laminin-111, HsLam-111 and HsLam-211 protein probed against α-human Laminin-111 **(C)** rod-domain and **(D)** C-terminal domain.

| \|  \| \| --- \| |  |
| --- | --- | --- |

| **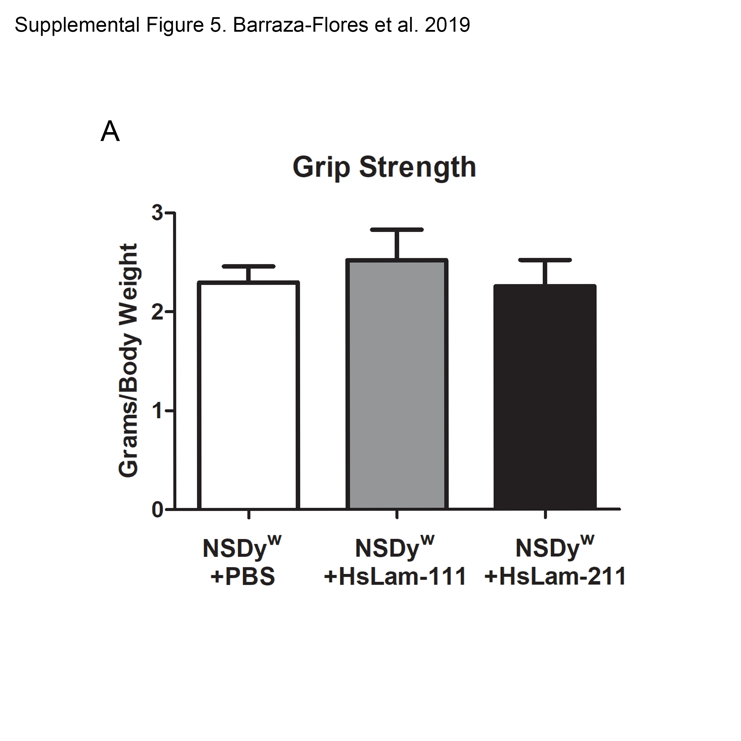** |
| --- |

Supplemental Fig 5. Treatment with human Laminin-111 or 211 does not significantly change grip strength in an immunocompromised mouse model of LAMA2-CMD. **(A)** HsLam-111 and HsLam-211 treated NSDy^W^ mice did not show a significant increase in grip strength when compared to PBS treated group (N=6).
